# Pneumocystis jirovecii and Nocardia pneumonia in a middle-aged male with Nephrotic syndrome: a case report and literature review

**DOI:** 10.1186/s12879-024-09987-6

**Published:** 2024-09-30

**Authors:** Yaqing Wang, Xiaojie He, Shuangyan Liu, Xiaodong Li

**Affiliations:** 1 Graduate School of Chengde Medical University, Chengde, Hebei 067000 China; 2 Graduate School of Hebei Medical University, Shijiazhuang, Hebei 050017 China; 3 Department of Nephrology, Baoding No 1 Central Hospital of Hebei Medical University, Baoding Great Wall North Street No 320, Baoding, Hebei 071000 China

**Keywords:** Nephrotic syndrome, Pulmonary infection, Co-infection, High-throughput nucleic acid sequencing

## Abstract

**Introduction:**

Nephrotic syndrome (NS) is a common chronic kidney disease that is often accompanied by a state of immunodeficiency. Immunosuppression increases the risk of infections, with Pneumocystis jirovecii and Nocardia brasiliensis being two opportunistic pathogens that can cause severe infections in patients with compromised immune function. This study presents a case of a middle-aged male patient with NS concurrently infected with Pneumocystis jirovecii and Nocardia brasiliensis. It aims to synthesize the pertinent diagnostic approaches and treatment experiences. Notably, there have been no reported cases of NS occurring simultaneously with both Pneumocystis jirovecii pneumonia and Nocardia pneumonia.

**Case presentation:**

A 58-year-old male farmer presented to the hospital with a one-week history of persistent fever, cough, and sputum production. His maximum body temperature was recorded at 39 °C, and he produced yellow viscous sputum. This patient had a one-year history of NS, managed with long-term oral corticosteroid and cyclophosphamide therapy. Admission chest computed tomography displayed interstitial changes in both lungs. After failing to detect any pathogens through routine etiological tests, we successfully identified Nocardia brasiliensis, Pneumocystis jirovecii, and Lodderomyces elongisporus using bronchoscopy-guided sputum samples through metagenomic next-generation sequencing (mNGS) technology. Subsequently, we initiated a combined treatment regimen for the patient using trimethoprim-sulfamethoxazole, meropenem, and moxifloxacin, which yielded remarkable therapeutic outcomes.

**Conclusion:**

The adoption and promotion of mNGS technologies have significantly resolved the difficulty in early pathogen detection, guiding clinicians from empirical to genomic diagnosis, achieving prevention before treatment, and thereby enhancing patient survival rates.

## Background

Pneumocystis jirovecii pneumonia (PJP) and Nocardiosis, opportunistic infections, primarily affect individuals with impaired immune systems [1–3]. Nephrotic syndrome (NS), as a prevalent etiological factor, is associated with diminished immunological function [4]. This condition results in proteinuria, leading to decreased serum protein levels and subsequent immunosuppression [5]. Both PJP and Nocardiosis present non-specific clinical manifestations, with diagnoses relying heavily on etiological testing [6, 7]. Recent advancements in high-throughput sequencing technologies have enhanced their use in infectious disease diagnosis, allowing for swift and accurate pathogen identification [8–11]. In particular, metagenomic next-generation sequencing (mNGS) enables direct sequencing of infectious samples, thus capturing and preserving extensive pathogenic data [9]. This method offers a rapid and precise diagnostic foundation for complex infections following corticosteroid and immunosuppressant use [8]. This study details a case of concurrent Pneumocystis jirovecii and Nocardia infection in an NS patient, highlighting the successful diagnostic application of mNGS technology. It seeks to compile clinical experiences and provide insights for the prompt and precise diagnosis, treatment, and prognosis of these diseases.

## Case presentation

**Fig. 1:**
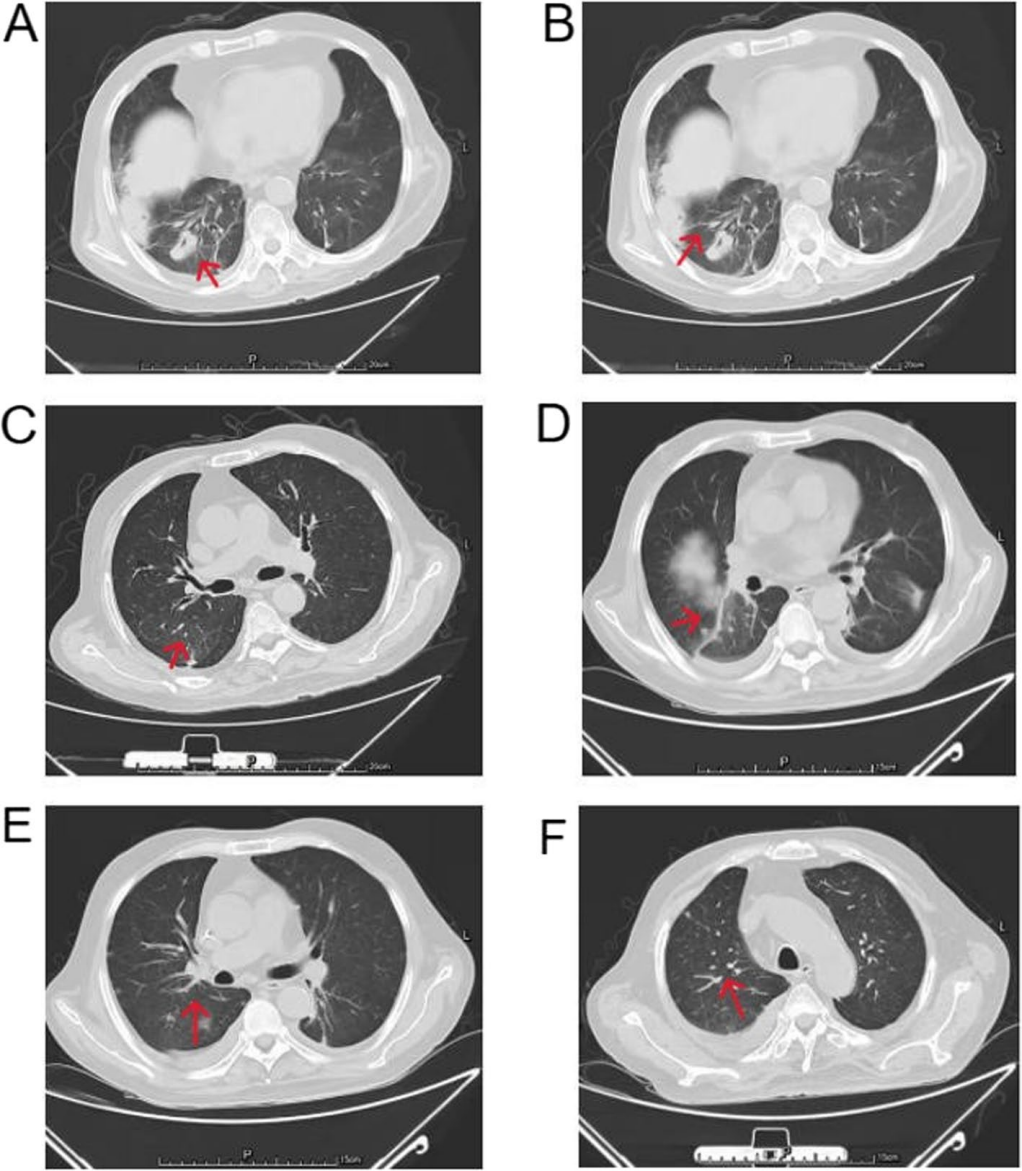
**A**-**C** Admission chest CT displayed interstitial changes in both lungs, a small amount of effusion in the right thoracic cavity, multiple small nodular and strip-like dense shadows. **D**-**F** Two weeks after admission chest CT showed a significant reduction in patchy shadows in both lungs

A 58-year-old male farmer presented to the hospital with a one-week history of persistent fever, cough, and sputum production. His maximum body temperature was recorded at 39 °C, and he produced yellow viscous sputum. This patient had a one-year history of NS, managed with long-term oral corticosteroid therapy (initial methylprednisolone dose: 24 mg) and cyclophosphamide pulse therapy (cumulative dose: 6.0 g). Upon admission, a complete blood count was performed, which showed elevated lymphocytes along with mild anemia. Lactate dehydrogenase, procalcitonin, and C-reactive protein (CRP) levels were found to be elevated, with a notably low CD4^+^ T lymphocyte percentage of 10.63% (normal range: 28.5%-60.5%). Quantitative assays for anti-neutrophil cytoplasmic antibodies, influenza A and B viruses, cytomegalovirus, and a range of respiratory pathogens, including Mycoplasma, Chlamydia, Coxsackie virus, respiratory syncytial virus, adenovirus, and Epstein-Barr virus, as well as tuberculosis interferon-gamma release assays, disclosed no significant findings. Details of the specific laboratory results are presented in Table 1. Admission chest computed tomography (CT) (Fig. 1, A-C) displayed interstitial changes in both lungs. Following a diagnosis of pulmonary infection (PI), this patient was administered meropenem (0.5 g) and moxifloxacin (0.4 g) via daily intravenous drips for infection management. This regimen was augmented by transfusions, expectorant therapy, and nutritional support.

**Table 1:** Lab examination in our hospital

Variable	Normal Range	Day 1	Day 5	Day 10	Day 15
WBC	4–10 (× 10^9^/L)	5.90	3.81	2.91	4.57
RBC	4.5–5.5(× 10^12^/L)	2.89	2.61	2.04	2.81
Platelet	100–300(× 10^9^/L)	150	149	121	78
Hb	110–150(g/L)	95	71	67	86
NEUT	1.8–6.3(g/L)	5.56	3.59	2.57	3.85
Lymphocyte	1.2–4.8(× 10^9^/L)	0.13	0.11	0.25	0.56
Urine WBC	0–5.4(/HPF)	0.79	/	/	/
Urine RBC	0–4.5(/HPF)	2.03	/	/	/
Urine Protein	-	4 +	/	/	/
24hUpros	0–0.15(g)	2.07	/	/	/
TP	65–85(g/L)	42.5	38.9	41.0	44.6
Alb	40–55(g/L)	23.6	15.0	18.90	18.8
LDH	120–250(U/L)	357.0	/	/	/
ALT	9–50(U/L)	62.0	10.9	14.2	9.90
AST	15–40(U/L)	70.2	11.9	25.1	13.7
Urea	3.6–9.5(mmol/L)	7.20	6.50	6.68	6.43
CREA	97–97 (umol/L)	57.0	59.3	106.7	77.1
Potassium	3.5–5.3(mmol/L)	4.19	3.22	4.21	4.24
Sodium	137–147(mmol/L)	138	142.5	136.9	143.7
Chlorine	99–110(mmol/L)	104	108.3	105.6	108.6
Calcium	2.11–2.52(mmol/L)	1.98	1.77	1.81	1.82
Phosphorus	0.85–1.51(mmol/L)	0.99	0.85	0.92	0.94
Magnesium	0.75–1.02(mmol/L)	0.73	0.77	0.81	0.78
PCT	0–0.25(ng/mL)	0.58	4.23	0.44	0.31
CRP	0–8(mmol/L)	188.0	102.0	101.0	92.0
C3	0.79–1.52(g/L)	1.53	/	/	1.05
C4	0.16–0.38(g/L)	0.29	/	/	0.25
IgA	1–4.2(g/L)	0.93	/	/	2.00
IgG	8.6–17.4(g/L)	3.19	/	/	4.95
IgM	0.3–2.2(g/L)	0.74	/	/	1.89
ESR	0–15(mm/h)	137	129	/	25
BDG	0–100.5(pg/ml)	89.14	/	/	/
GM	< 0.5S/CO	1.05	/	/	/

*WBC* White blood cell, *RBC* Red blood cell, *Hb* Haemoglobin, *NEUT* Neutrophils, *24hUpro* 24-h urine proteins, *TP* Total protein, Albalbumin, *LDH* Lactate dehydrogenase, *ALT* Alanine aminotransferase, *AST* Aspartate aminotransferase, *UREA* Urea-nitrogen, *CREA* Creatinine, *PCT* Procalcitonin, *CRP* C-reactive protein, *C3* Complement3, *C4* Complement4, *ESR* Erythrocyte sedimentation rate, *BDG 1* 3-β-d-Glucan, *GM* Galactomannan, ‘/’indicates missing data

**Fig. 2:**
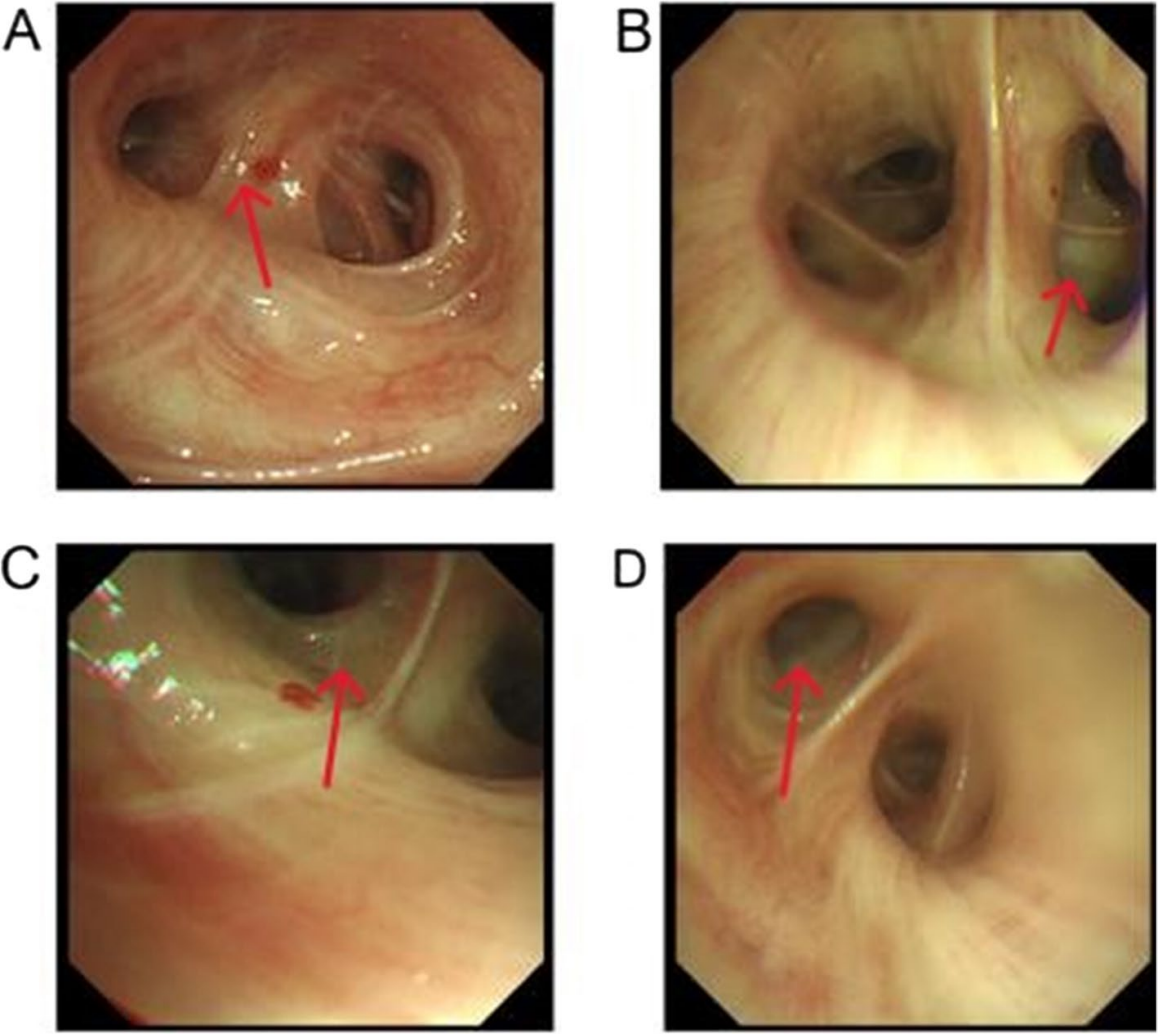
Fiberoptic bronchoscopy displayed inflammation within the airway, a large amount of yellow purulent sputum in the lumen of the right middle lobe bronchus

After five days into the preliminary antimicrobial treatment, the patient still presented with fever despite, negative sputum and blood cultures. The (1 → 3)-β-D-glucan test showed a level of 89.14 pg/mL (normal value: 0–100.05 pg/mL), and galactomannan for Aspergillus was 1.05 (normal value: < 0.5 S/CO). On the fifth day of admission, fiberoptic bronchoscopy (Fig. 2) disclosed airway inflammation. Cultures of the bronchoalveolar lavage fluid indicated an infection with Nocardia, and mNGS of the microbial metagenome from the lavage fluid detected Nocardia brasiliensis, Pneumocystis jirovecii, and Lodderomyces elongisporus. Specific test results can be found in Table 2. We performed a drug sensitivity assay on the bronchoalveolar lavage fluid employing the disk diffusion technique, revealing sensitivity to sulfonamides. Promptly, we reviewed pertinent literature and sought consultation from infectious disease specialists [2]. Based on their recommendations, this patient received oral co-trimoxazole (sulfamethoxazole 0.4g4 and trimethoprim 80mg4, three times daily) and administered human albumin to enhance his immune function. After two weeks of medical interventions, there was a significant improvement in the patient's overall condition. Chest CT (Fig. 1, D-F) showed a reduction in pulmonary patchy shadows. The patient's infectious indicators markedly improved, and symptoms including cough, sputum production, and fever substantially abated too. Thus, he was discharged. He was instructed to persist with co-trimoxazole therapy post-discharge and engage in regular outpatient follow-ups. Four weeks post-discharge, the patient revisited our outpatient clinic for a follow-up and his condition was stable without any symptoms of fever, cough, or expectoration.

**Table 2:** High throughput sequencing results of pathogenic microorganisms

Genus		Species		Relative abundance
Name	Number of detected sequences	Name	Number of detected sequences	
Nocardia	425898	Nocardia brasiliensis	189131	5.77%
Pneumocystis	5	Pneumocystis jiroveii	5	1.48%
Lodderomyces	3	Lodderomyces elongisporus	3	0.51%

1. Nocardia brasiliensis: This bacterium belongs to the genus Nocardia and can cause nocardiosis and mycetoma

2. Pneumocystis jirovecii: This organism is an opportunistic pathogen that causes Pneumocystis pneumonia. It primarily affects individuals with weakened immune systems

3. Lodderomyces elongisporus: This yeast can produce sporangiospores and can cause fungal infections such as fungemia and endocarditis in humans

## Discussion

NS is a common renal affliction characterized principally by glomerular filtration dysfunction, proteinuria, and histopathological damage to renal tissues [4]. Patients with this condition exhibit reduced serum immunoglobulin G and complement levels, leading to suppressed T-cell immunity, rendering them more susceptible to pathogenic microorganisms [12]. PJP is an opportunistic respiratory infection that occurs in preterm infants, organ transplant or cancer patients, individuals with immune deficiencies, and those receiving radiotherapy or immunosuppressive drugs [13]. Pneumocystis predominantly colonizes the lungs' alveoli, attaching to the alveolar epithelium. A deficiency or dysfunction of alveolar macrophages can precipitate infection [14]. The clinical manifestations include fever, cough, dyspnea, and chest pain, with rapid progression and high mortality rates [15]. Nocardia pneumonia is a rare opportunistic infection, contracted through inhalation of spores present in soil, water sources, and animal feces, typically occurring in the context of chronic or long-term glucocorticoid treatment and cellular immune deficiencies. Prolonged glucocorticoid use represents a significant risk factor for Nocardia infection as it inhibits Th1 cell-mediated immunity [14].

PJP and Nocardia pneumonia are two rare infectious diseases that we will explore in conjunction with NS. The kidneys serve as crucial excretory organs, but NS impairs their filtration function, particularly the glomerular filtration rate, which suppresses the immune system and reduces resistance to pathogens. Patients afflicted with NS experience diminished serum immunoglobulin G and complement levels [4]. Moreover, pharmacotherapy may represent an additional contributing factor to these infections, as patients constantly undergo treatments with glucocorticoids and immunosuppressive drugs, which decrease the body's defenses against pathogens and increase the risk of infection [5]. Prolonged contact with soil also makes concurrent infection with Pneumocystis jirovecii and Nocardia highly probable.

Diagnostic adjuncts play a pivotal role in disease confirmation. Upon patient admission, we conducted a suite of laboratory and radiological assessments to investigate the pathophysiological alterations. Inflammatory markers, such as CRP and erythrocyte sedimentation rate were elevated, indicating an inflammatory response. Chest CT revealed interstitial alterations in both lungs, scant right-sided pleural effusion, numerous minute nodular opacities, and linear densities, consistent with the radiological signature of Pneumocystis and Nocardia infections [15, 16]. The identification of Nocardia from bronchoalveolar lavage fluid cultures confirmed the presence of a rare bacterial infection. Furthermore, mNGS technologies detected Nocardia, Pneumocystis jirovecii, and Lodderomyces elongisporus in the lavage fluid. Lodderomyces elongisporus is a rare and emerging pathogen capable of causing fungemia and fungal endocarditis. However, due to its extremely low gene abundance detected by mNGS and the absence of any clinical symptoms in the patient attributed to this organism, we have excluded it as a potential pathogen. While mNGS exhibits higher sensitivity compared to traditional culturing methods, enabling the revelation of etiological information on potential polymicrobial infections and significantly improving the diagnostic accuracy of pneumonia, it still poses a significant cost burden and has limitations in that it cannot unequivocally distinguish whether the detected microorganisms are truly pathogens [9–11].

Patients with PJP exhibit non-specific clinical symptoms, predominantly characterized by progressive dyspnea. Temperature elevation is often moderate to mild fever, and the condition progresses rapidly. The principal clinical manifestations of Nocardia pneumonia include coughing, purulent or bloody sputum production, accompanied by symptoms resembling tuberculous intoxication, such as fever, night sweats, and weight loss. Radiological examinations may reveal a variety of presentations, including nodules, extensive infiltrates, consolidation, masses, and cavities, which may be associated with abscesses and pleural effusion [17, 18]. The clinical presentation of the patient is characterized by pyrexia, with body temperatures reaching up to 39 °C, subsequently accompanied by coughing, expectoration, and symptoms of fatigue. Chest CT imaging results revealed bilateral pulmonary inflammation, further corroborating the diagnosis of a PI.

For such co-infections, we have considered antimicrobial treatment, supportive care, and etiologic therapy in our comprehensive management approach. Both Pneumocystis jirovecii and Nocardia species exhibit drug resistance, rendering the selection of appropriate antimicrobial agent’s paramount. Presently, Trimethoprim-Sulfamethoxazole is the drug of choice for treating infections by these organisms, with a recommended treatment duration of 14 days. However, this may be extended based on clinical judgment [19]. For our patient, treatment with Trimethoprim-Sulfamethoxazole (sulfamethoxazole 0.4 g*4 and trimethoprim 80 mg*4, three times daily) was administered, coupled with bronchoscopy-guided bronchoalveolar lavage and bronchoscopic sputum suctioning to alleviate symptoms of cough and expectoration. Tang H and colleagues have identified the benefits of flexible bronchoscopy in relieving respiratory symptoms, promoting lesion absorption, and improving clinical outcomes in patients with severe pneumonia [20]. It is imperative to note that immune suppressants must be discontinued during the treatment process, and if necessary, immunostimulants may be utilized to enhance immune function. Furthermore, symptomatic and supportive treatments are crucial, such as administering oxygen therapy appropriately based on the patient's clinical symptoms and arterial blood gas analysis results.

A thorough search was performed across multiple online databases including PubMed, Embase, and Web of Science using the search terms “Pneumocystis jirovecii pneumonia” AND “Nocardiosis”. We reviewed the retrieved articles, examined the abstracts of all retrieved articles, and conducted data extraction and analysis. The inclusion criteria for the literature were: (1) case reports of co-infection with Pneumocystis jirovecii and Nocardia pneumonia published between January 1, 1986, and December 31, 2023; (2) articles describing definitive etiological evidence of Pneumocystis jirovecii and Nocardia pneumonia based on pathology and culture evidence. To date, there have been seven reported cases of co-infection with Pneumocystis jirovecii and Nocardia pneumonia, with the primary symptoms being fever, cough, and dyspnea. Among these cases, three were complicated by Acquired Immune Deficiency Syndrome (AIDS), one by smoldering adult T-cell leukemia, and the remaining three were administered glucocorticoids and immunosuppressive therapy for heart transplantation, dermatomyositis, and herpes, respectively (Table 3). Particularly noteworthy is the case reported by Tomohiko Koibuchi et al., where a patient with concurrent AIDS succumbed to respiratory failure, [24] while the other six patients experienced favorable therapeutic outcomes. The patient we reported underwent definitive pathogen identification through mNGS technology, and subsequent timely administration of co-trimoxazole treatment led to a significant improvement in the patient's condition. This underscores the paramount importance of early and prompt detection of causative infectious agents in clinical practice, as it holds pivotal significance in guiding both diagnosis and treatment. Thus, the patient we report herein represents the first case of PJP and Nocardia pneumonia co-infection detected through mNGS technology, concurrently complicated by NS.

**Table 3:** Analysis of demographic and clinical characteristics of 7 cases of mixed infection with Pneumocystis jirovecii and Nocardia pneumonia

First author/Year	Location	Number	Age/ Gender	Risk factor/comorbidity	Method of identification	Infection signs/ symptoms	Treatment	Prognosis
J L Rodriguez [21] /1986	America	1	46/Male	AIDS	BAL	Fever, diarrhea	Minocycline	Survive
H Perschak [22] /1991	Switzerland	2	63/Not mention	heart transplantation, glucocorticoids and immunosuppressants	Sputum culture BAL	Fever, dyspnea	TMP/SMX	Survive
			49/Male	Dermatomyositis, glucocorticoids and immunosuppressants	BAL	Fever	TMP/SMX	Survive
I Owan [23] /1994	Japan	1	76/Male	smoldering adult T-cell leukemia	Sputumculture BAL	Fever	Cefuzonam, T compounds	Survive
Tomohiko Koibuchi [24] /2002	Japan	1	27/Male	AIDS	Sputum culture BAL	Fever, dyspnea	Pentamidine isethionate	Death
Sohei Harada 1 [25] /2009	Japan	1	79/Male	bullous pemphigoid, glucocorticoids and immunosuppressants	Sputum culture	Fever	IMP, AMP, pentamidine	Survive
Kentaro Imai [26] /2011	Japan	1	33/Male	AIDS	Sputum culture	Fever, cough	IMP, AMP, garenoxacin	Survive

*AIDS* AcquiredImmune Deficiency Syndrome, *BAL* Bronchoalveolar lavage, *TMP* Trimethoprim, *SMX* Sulfamethoxazole, *IMP* Imipenem, AMK Amikacin

## Conclusion

To our knowledge, this is the first reported case of NS co-occurring with PJP and Nocardia pneumonia. For clinicians, the diagnosis and treatment of NS patients who are undergoing immunosuppressive therapy and have concurrent PI represent a formidable challenge. Prompt diagnosis of PI is crucial for improving patient prognosis and reducing the incidence of complications. Early pathogen detection can optimize the use of antibiotics and reduce hospitalization time. The adoption and promotion of mNGS technologies have significantly resolved the difficulty in early pathogen detection. The widespread clinical application of mNGS has opened new vistas for the diagnosis of NS complicated by PI, guiding clinicians from empirical to genomic diagnosis, achieving prevention before treatment, and thereby enhancing patient survival rates.

## Data Availability

The original contributions presented in the study are included in the article,further inquiries can be directed to the corresponding author.

## Abbreviations

NS: Nephrotic syndrome
mNGS: Metagenomic next-generation sequencing
PJP: Pneumocystis jirovecii pneumonia
CT: Computed tomography
CRP: C-reactive protein
PI: Pulmonary infection
AIDS: AcquiredImmune Deficiency Syndrome
BAL: Bronchoalveolar lavage
TMP: Trimethoprim
SMX: Sulfamethoxazole
IMP: Imipenem
AMK: Amikacin
